# Fibrodysplasia ossificans progressiva

**DOI:** 10.1002/ccr3.8165

**Published:** 2023-11-06

**Authors:** Chané Smit, André Uys

**Affiliations:** ^1^ Department of Oral and Maxillofacial Pathology, School of Dentistry University of Pretoria Pretoria South Africa; ^2^ Department of Anatomy, School of Medicine University of Pretoria Pretoria South Africa

**Keywords:** cone‐beam computed tomography, heterotopic ossification

## Abstract

**Key Clinical Message:**

Fibrodysplasia ossificans progressiva is a progressively debilitating condition associated with significant morbidity caused by heterotopic ossification. Recognition of the early signs of hallux valgus and painful soft tissue nodules can assist in the early diagnosis of this condition. Periodic radiographic examination is mandatory to monitor the disease progression.

**Abstract:**

Fibrodysplasia ossificans progressiva is a rare condition with an estimated prevalence of one in two million individuals. The condition is characterized by widespread heterotrophic ossification of skeletal muscles and ligaments. We report the case of an 8‐year‐old female patient and show the radiological progression of the condition.

## 1 INTRODUCTION

Heterotopic ossification is associated with ectopic bone formation within soft tissues such as skeletal muscles, cartilage, tendons, ligaments, and fascia. Fibrodysplasia ossificans progressiva (FOP) is a rare condition characterized by widespread heterotrophic ossification and is caused by a mutation in the bone morphogenetic protein (BMP) Type 1 receptor, activin receptor 1A/activin‐like kinase 2 (ACVR1/ALK2).^1^ All the identified causative mutation sites of FOP are located in either the GS‐rich domain or the serine/threonine kinase domain.^2^ Fibrodysplasia ossificans progressiva can be inherited in an autosomal dominant pattern or can occur sporadically. The only method to establish FOP at a molecular level is by DNA sequence analysis of the ACVR1 gene. Studies indicate ACVR1^R206H^ mutations in approximately 97% of patients presenting with FOP.^3^ Additional pathogenic variants in ACVR1 have been identified and include ACVR1^R375P^ and ACVR1^G356D^.^3^, ^4^ Initial presenting symptoms include bilateral hallux valgus and painful soft tissue nodules on the back and neck.^5^ A defining clinical feature of FOP is malformation of the big toe with a prevalence of more than 95%.^6^, ^7^ Ultimately, the extensive heterotopic ossification leads to impaired mobility and is referred to as the so‐called “Stoneman's disease.”^8^

## 2 CASE REPORT

**FIGURE 1:**
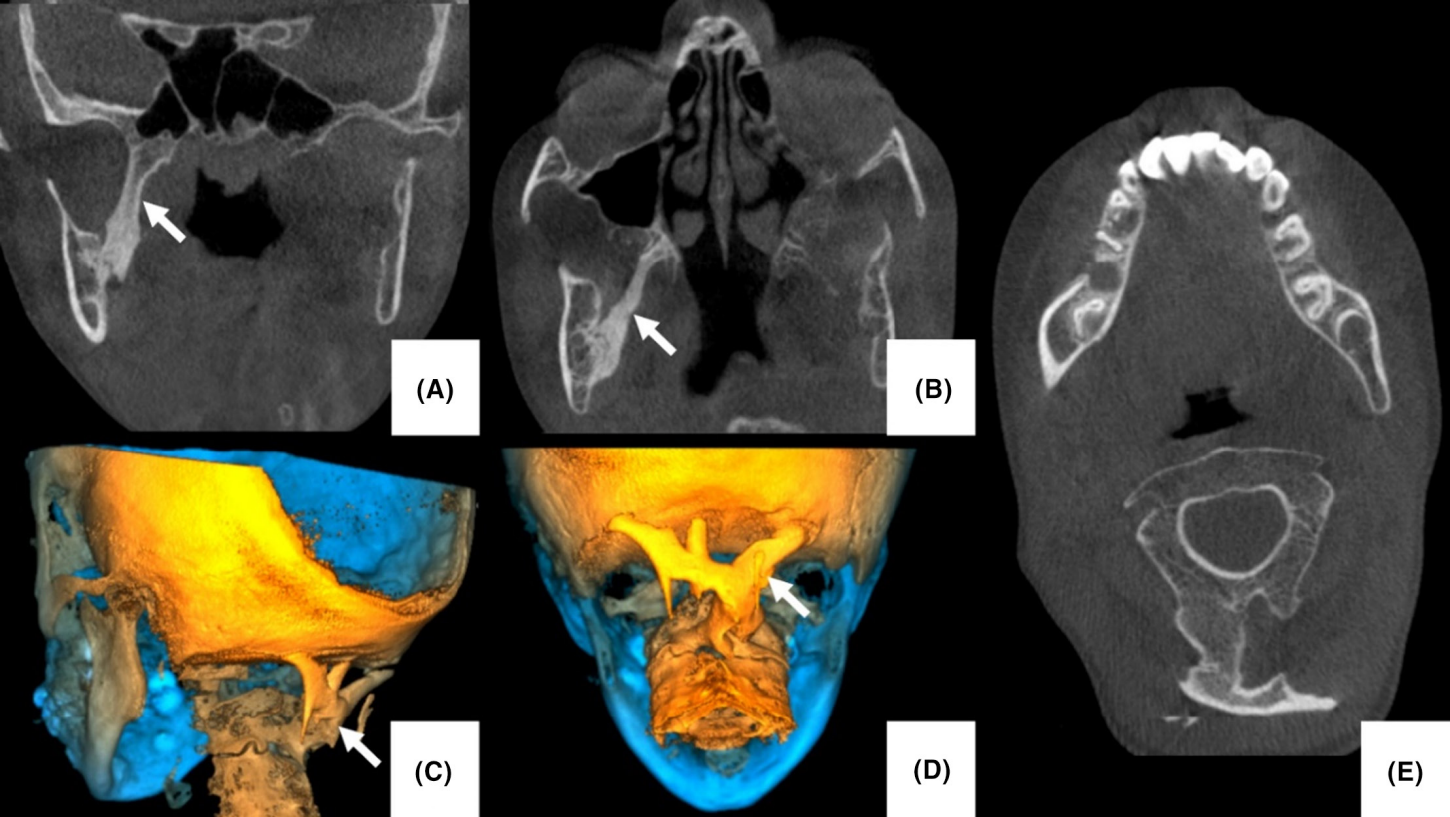
(A) Coronal and (B) axial CBCT images showing calcification of the right medial pterygoid muscle (arrows). (C, D) Three‐dimensional reconstructions and (E) axial CBCT images showed calcification of the rectus capitus muscles (arrows) as well as a decreased space between the cervical vertebrae.

An 8‐year‐old female patient presented to the University of Pretoria Oral Health centre with progressive limb stiffness and loss of mobility. She was referred due to limited mouth opening and trismus. She was diagnosed with fibrodysplasia ossificans progressiva at 2 years with no other members of the family presenting with similar symptoms. Cone‐beam computed tomography (CBCT) examination revealed ossification of the medial pterygoid muscle on the right (Figure 1A,B) as well as the rectus capitus muscles (Figure 1C–E). Decreased articular space between the cervical vertebrae was also noted.

**FIGURE 2:**
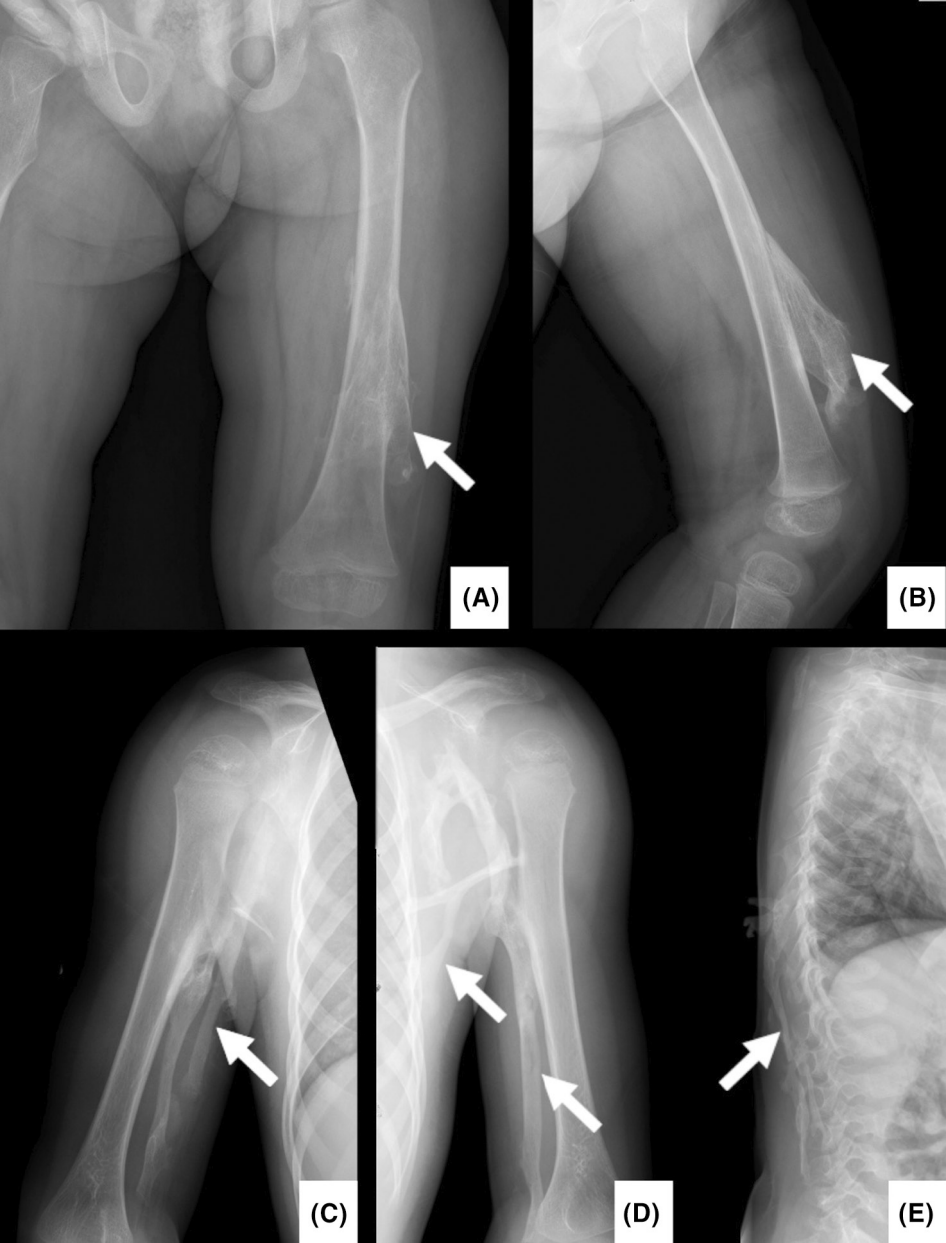
(A) Posterior–anterior and (B) lateral radiographs of the left leg showing ossification of the vastus lateralis muscle (arrows). Posterior–anterior radiographs of the (C) right and (D) left arm showing calcifications of the bicep muscles and bridging ossification with the lateral chest wall. (E) lateral spine radiograph showing ossifications of the erectus spinae muscles (arrow).

**FIGURE 3:**
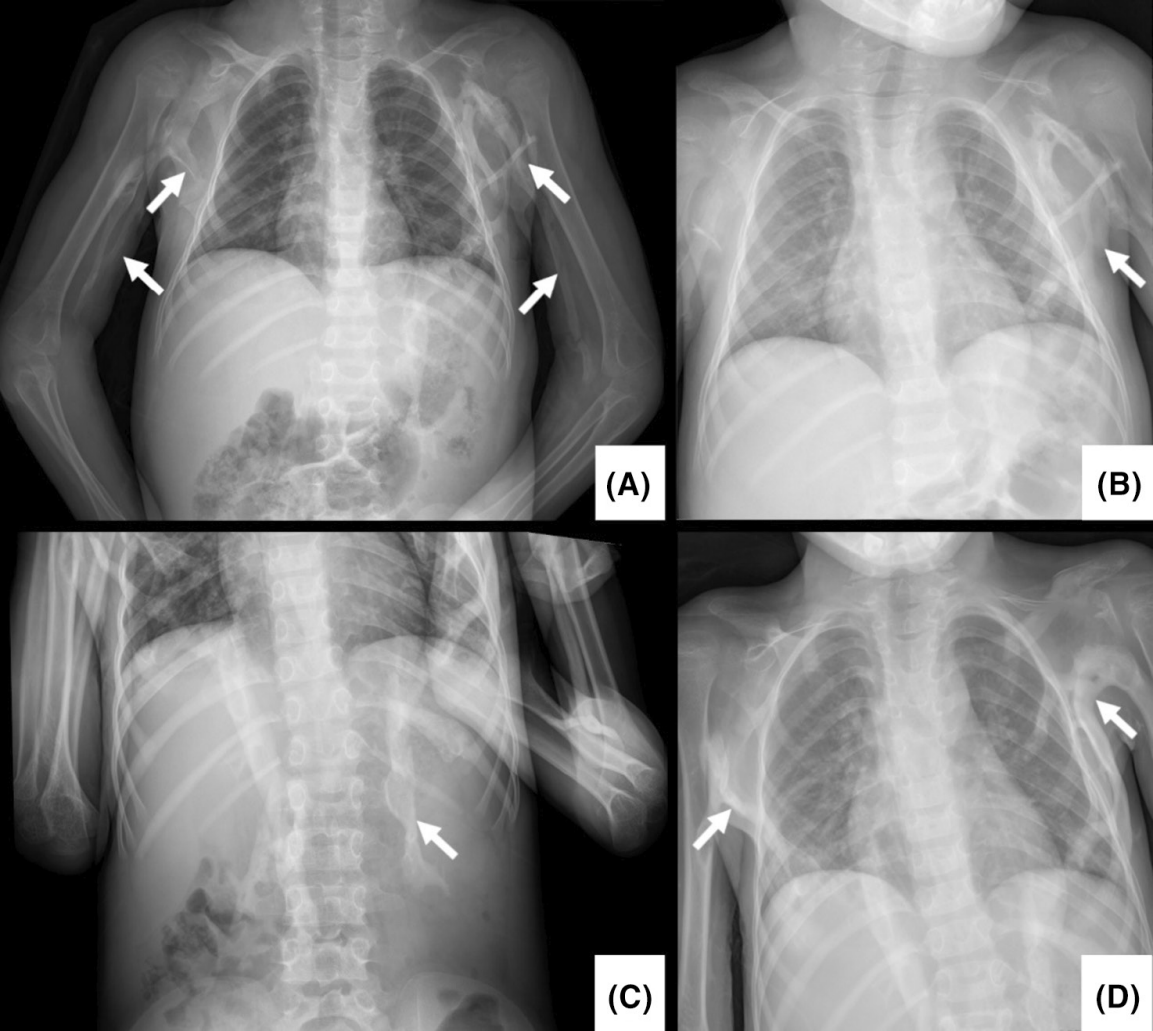
(A, B) Posterior–anterior chest radiographs when the patient was 2‐year‐old showing ossification in the biceps (inferior arrows in A) and pectoralis major (superior arrows in A and arrow in B) as well as decreased articular spaces between the cervical vertebrae. (C, D) Posterior–anterior chest radiographs when the patient was 6‐year‐old showed progression in the calcifications in the axillae and calcifications adjacent to the spine (erectus spinae).

Previous radiographic examinations at 2 years of age, revealed calcification of muscles involving the left leg (Figure 2A,B), left and right arm (Figure 2C,D) as well as the spine (Figure 2E). Four years later when the patient was 6 years old a barium swallow and another spine and a chest X‐ray were performed. The radiographs revealed progressive ossification in the axilla, arms and spine when the girl was 2 years (Figure 3A,B) compared to 6 years of age (Figure 3C,D).

Due to a language barrier, limited communication was possible. No lab records of any blood tests could be traced and the patient was lost to follow‐up.

## 3 DISCUSSION

Fibrodysplasia ossificans progressiva is extremely rare with an estimated prevalence of one in two million individuals.^9^ The mean age of FOP diagnosis is at the age of 7 years with almost all patients presenting with loss of mobility at this early stage.^8^, ^10^ Patients often present with initial symptoms of a malformed first metatarsal (hallux valgus) and painful soft tissue, sub‐facial nodules on the back and neck that later ossify.^5^ The clinical course often starts with a painful soft tissue swelling followed by ossification that results in loss of mobility and stiffness. Clinical signs often start earliest in the neck and upper back followed by the lower back, shoulders, and chest.^10^ The progression of ossification commonly follows the same pattern as embryonic skeletal development.^11^ Fibrodysplasia ossificans progressiva can present with different symptoms in different age groups and become progressively worse with age.^5^ Episodic flare‐ups of FOP can occur 2–6 times a year and present as soft tissue swellings, pain and loss of mobility followed by progressive heterotopic ossifications.^10^ The flare‐up episode can occur sporadically or after minor trauma.^10^ Patients can present with variable symptoms including, large spinous processes, temporomandibular joint malformations, and fusion of cervical vertebrae.^5^, ^9^, ^12^ The diaphragm, cardiac muscles, tongue, and ocular muscles are often spared.^9^, ^11^ Although the major skeletal abnormalities of FOP are extensively published, the non‐skeletal manifestations such as neurologic and cardiopulmonary disruptions are often more concerning.^13^

In most cases, the clinical and radiological signs are diagnostic for FOP but genetic testing can be performed. An increased alkaline phosphatase level can also be detected.^5^ Radiological evaluation is not only necessary for accurate diagnosis but also to monitor disease progression. Plain film radiographs are an inexpensive and relatively low‐dose method to detect which muscles are affected but is limited in detecting lesions in the early inflammatory phase.^12^ Magnetic resonance imaging (MRI) or ultrasound (US) is useful in detecting soft tissue swelling and lesions in the early or flare‐up phases.^12^ Computed tomography (CT) imaging is useful in evaluating the extent, volume and progression of oedema and calcification to ossification of affected tissues.^12^ Bone scintigraphy and positron emission tomography (PET) can also assist in the detection of heterotrophic ossifications.^11^

Treatment guidelines have still not been established as biopsy or surgical intervention often leads to the progression in ossification. Non‐interventional treatment consisting of avoidance of trauma, physiotherapy in combination with non‐steroidal anti‐inflammatory or cyclo‐oxygenase inhibitors during flare‐up episodes, has been advocated.^14^ Surgical intervention with removal of debilitating lesions has shown variable outcomes and is usually only advocated in extreme cases.^14^ Research into therapeutic advances to limit the disabling effects of this condition is currently conducted, giving hope for possible improved outcomes.^1^, ^15^ Multiple potential therapeutic targets, based on the underlying molecular mechanism for FOP, have been identified for drug development. Development is focusing on small‐molecular inhibitors and antibodies targeting ALK2. Clinical trials are currently underway and drugs such as, saracatinib, INCB000928 DS‐6016a, BLU‐782 (IPN60130), REGN2477 (garetosmab), rapamycin, and palovarotene are being tested for FOP treatment.^2^

## AUTHOR CONTRIBUTIONS

**Chané Smit:** Conceptualization; investigation; methodology; writing – original draft. **André Uys:** Conceptualization; investigation; methodology; writing – review and editing.

## FUNDING INFORMATION

This research did not receive any specific grant from funding agencies in the public, commercial, or not‐for‐profit sectors.

## CONFLICT OF INTEREST STATEMENT

The authors declare that they have no conflict of interest.

## ETHICS STATEMENT

This study was approved by the University of Pretoria, Faculty of Health Sciences Research Ethics Committee (Reference no.: 139/2022). All procedures followed the ethical standards of the Helsinki Declaration of 1975, as revised in 2008.

## CONSENT

The written patient consent has been signed and collected in accordance with the journal's patient consent policy.

## Data Availability

The data that support the findings of this study are available from the corresponding author upon reasonable request.
